# Three Phase-Grating Moiré Neutron Interferometer for Large Interferometer Area Applications

**DOI:** 10.1103/PhysRevLett.120.113201

**Published:** 2018-03-16

**Authors:** D. Sarenac, D. A. Pushin, M. G. Huber, D. S. Hussey, H. Miao, M. Arif, D. G. Cory, A. D. Cronin, B. Heacock, D. L. Jacobson, J. M. LaManna, H. Wen

**Affiliations:** 1 Department of Physics, University of Waterloo, Waterloo, Ontario, Canada N2L3G1; 2 Institute for Quantum Computing, University of Waterloo, Waterloo, Ontario, Canada N2L3G1; 3 National Institute of Standards and Technology, Gaithersburg, Maryland 20899, USA; 4 Biophysics and Biochemistry Center, National Heart, Lung and Blood Institute, National Institutes of Health, Bethesda, Maryland 20892, USA; 5 Department of Chemistry, University of Waterloo, Waterloo, Ontario, Canada N2L3G1; 6 Perimeter Institute for Theoretical Physics, Waterloo, Ontario, Canada N2L2Y5; 7 Canadian Institute for Advanced Research, Toronto, Ontario, Canada M5G1Z8; 8 University of Arizona, Department of Physics, Tucson, Arizona 85721, USA; 9 Department of Physics, North Carolina State University, Raleigh, North Carolina 27695, USA; 10 Triangle Universities Nuclear Laboratory, Durham, North Carolina 27708, USA

## Abstract

We demonstrate a three phase-grating moiré neutron interferometer in a highly intense neutron beam as a robust candidate for large area interferometry applications and for the characterization of materials. This novel far-field moiré technique allows for broad wavelength acceptance and relaxed requirements related to fabrication and alignment, thus circumventing the main obstacles associated with perfect crystal neutron interferometry. We observed interference fringes with an interferometer length of 4 m and examined the effects of an aluminum 6061 alloy sample on the coherence of the system. Experiments to measure the autocorrelation length of samples and the universal gravitational constant are proposed and discussed.

Interferometers employing particle self-interference have proven to be an extremely sensitive measuring tool, allowing for the precise characterization of material properties as well as measurements of fundamental constants [1,2]. Neutrons, in particular, are a convenient probe due to their relatively large mass, electric neutrality, and subnanometer-sized wavelengths. The earliest neutron interferometer (NI) was formed via a pair of prisms and, through wave front division, achieved Fresnel interference effects with up to 60 *μ*m path separations [3]. Amplitude division from Bragg diffraction off of crystal planes was later used to make perfect crystal NIs with Mach-Zehnder (MZ) path separations of several centimeters [4]. This relatively large path separation along with the macroscopic size of the interferometer contributed to its success in exploring the nature of the neutron and its interactions [5–10]. However, perfect crystal NIs possess a very narrow wavelength acceptance, are difficult to fabricate, and operate only under stringent forms of vibration isolation and beam collimation [11–15]. This limits their widespread adaption at many neutron sources.

Microfabricated periodic structures have been employed as neutron optical elements to produce quantum interference. This led to a demonstration of a MZ -based grating NI with reflection gratings [16] and a three phase-grating MZ NI for low energy (*<*1 meV) neutrons [17–20]. However, the inherently low intensity of *<*1 meV neutrons makes it difficult for these grating interferometers to outperform the perfect crystal NI.

Here, we demonstrate a broadband, three phase-grating moire interferometer (PGMI) operating in the far field. The schematic diagram of the setup is depicted in Fig. 1(a). The three PGMI employs the universal moiré effect [21] and is an extension to the recently demonstrated two phase-grating moiré neutron interferometer [22,23]. Contrary to the typical MZ interferometers that have two separate and distinct beam paths, the PGMI works in the full field of a cone beam from a finite source, similar to in-line holographic devices. Such full-field systems can be understood intuitively in the framework of Fourier imaging developed by Cowley and Moodie [24,25]: the second grating produces a series of achromatic Fourier images of the first grating at a specific “echo plane” downstream. The third grating is detuned from the echo plane to produce a phase moiré effect with the Fourier images, which is observed as a beat pattern in intensity in the far field. When all three gratings have the same period (*λ*_*G*_) the fringe period at the detector (*λ*_*d*_) is given by [21]

(1) $$\lambda_{d}=\frac{\left(L_{1}+D_{1}+D_{2}+L_{2}\right)}{\left|D_{2}-D_{1}\right|}\lambda_{G},$$

where *L*_1_, *D*_1_, *D*_2_, and *L*_2_ are defined in Fig. 1(a). The fringe frequency at the detector is given by *f*_*d*_ = 1/*λ*_*d*_. The Fourier image and the third phase grating both possess regular square grating profiles. Therefore, the moiré pattern they create are broad straight fringes. If there is an angle between the two, the direction of the moiré fringes will rotate relative to the grating direction of the third grating. A more detailed description of the interferometer’s functionality can be found in the Supplemental Material [26].

The main differences between the three PGMI and the neutron Talbot-Lau grating interferometer [27,28] are that only phase gratings are used, a broader wavelength distribution is accepted, and the fringes are observed in the far field. The phase moiré effect in the far field produces large period interference fringes that are orders of magnitude larger than the period of the gratings, enabling direct detection with an imaging detector without the need for an absorbing analyzer grating. Thus, a greater intensity is transmitted through a PGMI and the PGMI has comparatively relaxed grating fabrication requirements, especially at smaller grating periods. In addition, grating alignment for the PGMI is an order of magnitude less stringent compared to the perfect crystal NI, whose individual diffracting elements must be aligned relative to each other to within 0.01 arcsec [29]. Other advantages of this setup include the use of widely available thermal and cold neutron beams, large, variable interferometer area, and the broad, simultaneous wavelength acceptance.

The experiments were performed at the Cold Neutron Imaging (CNI) facility [30] at the National Institute of Standards and Technology’s (NIST) Center for Neutron Research, where a 20 MW reactor provides a steady flux of thermal and cold neutrons for a variety of instruments. The CNI is located at the end position of neutron guide 6 and as such has a polychromatic neutron spectrum that is approximately given by a Maxwell-Boltzmann distribution with *T*_*c*_ = 40 K or *λ*_*c*_ = 0.5 nm.

For this demonstration, we used Si gratings that were available, but not necessarily optimal for our setup. The period of each grating was 2.4 *μ*m. The first and third gratings had a depth of 16 *μ*m corresponding to a phase shift of ∼*π*/2 for the mean wavelength of 0.5 nm, while the second grating had a depth of 30 *μ*m corresponding to a phase shift of ∼*π*. The effect of a single *π*/2 or *π* phase grating on the neutron transverse momentum distribution is shown in Fig. 1(b). The gratings were oriented vertically to avoid beam deviation due to gravity. Rotational alignment of the gratings about the *z* axis was done with 0.01° accuracy. The slit width was set to 500 *μ*m and slit height to 1.5 cm. The slit to detector length was fixed at *L* = 8.8 m, while the distance between the slit and the second grating was fixed at 4.75 m. The detector used was a scientific CMOS camera viewing a 150-*μ*m-thick LiF:ZnS scintillator and had a spatial resolution of ∼150 *μ*m. The exposure time was 20 s per image, and the detector efficiency was ∼0.4.

The integrated [along the *x* direction in Fig. 1(a)] intensity profile recorded by the camera can be fit to a cosine function

(2) $$I=A+B\cos\left(2\pi f_{d}y+\phi \right),$$

where *y* is the pixel location on the camera, *A* is the mean, *B* is the amplitude, and *ϕ* is the differential phase. The contrast or “fringe visibility” is given by

(3) $$C=\frac{\max\left\{I\right\}-\min\left\{I\right\}}{\max\left\{I\right\}+\min\left\{I\right\}}=\frac{B}{A}.$$

Contrast as a function of the difference between the grating separations (*D*_2_ − *D*_1_) is plotted in Fig. 2. The distance between the first and second grating was *D*_1_ = 4.6 cm, while the distance between second and third grating *D*_2_ was scanned. In the Methods section of [21], it is shown that the contrast is dependent on the autocorrelation functions of the first and third grating profiles and that, for ideal 50% comb-fraction gratings, the contrast peaks when the autocorrelation distances are half the grating period. For our geometry, *D*_2_ − *D*_1_ ≈ 土1.2 cm values produce autocorrelation distances close to half a period for both first and third gratings. The peak positions of the observed contrast agree with this prediction. At equal separation distances, *D*_1_ = *D*_2_, the third grating is at the Fourier image location and no fringes are expected. On Fig. 2, it can also be seen that the fringe frequency is linearly proportional to the difference of separation distances, as per Eq. (1).

The first and the third grating can be translated away from the middle grating in synchronized intervals in order to achieve large interferometer length. Figure 3 shows the peak contrast as a function of the distance from the first to the third grating. The data are the contrast of the empty interferometer and illustrate how the contrast varies with the length of the interferometer, possibly pointing to coherence loss from increased air scattering and mechanical vibrations [31]. At each new length, the contrast optimization was performed by finely translating the third grating. Contrast was observed with an interferometer length of 4 m, which was the limit of our experimental setup. An interferometer area of ∼8 cm^2^ is estimated for that configuration.

To observe phase shifts inside the three PGMI and quantify the robustness of the setup, we performed “linear phase stepping,” depicted in Fig. 4. This process verifies that the phase shifts of the fringes from the grating movement agree with expected phase stepping behavior—movement of the grating by one period (2.4 *μ*m) causes a 2*π* phase shift. Here the phase shift of the induced interference fringes is obtained by parallel translation of the third grating with step sizes smaller than the period of the gratings. It was observed that the phase of the interference fringes linearly increases as expected.

Placing a sample between the gratings allows for phase and dark-field imaging [22,32]. For a rectangular sample of 6061 aluminum alloy, the linear attenuation of integrated intensity calculated as - $\ln\left(I_{\text{sample}}/I_{\text{empty}}\right)$ and the normalized contrast calculated as $C_{\text{sample}}/C_{\text{empty}}$ are shown in Fig. 5. It is observed that the sample degrades the relative contrast to 0.28, most likely due to small angle neutron scattering off of the microstructure present in the alloy. The images were obtained by the harmonic analysis method described in [33].

The three PGMI presents a unique opportunity for material characterization, as one can readily vary by orders of magnitude the autocorrelation length used to probe the sample. The method is analogous to the probing of autocorrelation lengths with a two phase-grating interferometer [23], but the probed autocorrelation length in this case is the separation length of the individual MZ interferometers depicted in Fig. 1(a),

(4) $$\Delta h\approx \frac{\lambda }{\lambda_{G}}L_{s},$$

where *L*_*s*_ is the distance from the first grating to the sample. Therefore, the unique ability of the three PGMI is accessing larger autocorrelation lengths (*>*100 *μ*m), which are beyond the standard limits of the ultrasmall angle neutron scattering and other neutron dark-field imaging methods. Potential applications would be the probing of porous mineral samples, oil or gas core samples, and man-made porous scaffolds and materials.

The enclosed area of the interfering neutron paths is an important parameter of a NI and its response to potential gradients and forces. Perfect crystal interferometers are limited to the practical size of commercially available perfect or dislocation free Si ingots. For perfect crystals with Bragg angles of ∼*π*/4, an area of ∼100 cm^2^ can be achieved for the particular monochromatic wavelength [15]. The three PGMI has the unique opportunity to reach and surpass the perfect crystal NI in this regard. In the current setup, with 2 m separation between the gratings, the enclosed area is ∼8 cm^2^ for 0.5 nm wavelength neutrons, while it is ∼15 cm^2^ for largest perfect crystal NI available at NIST for 0.271 nm neutrons. Reducing the grating period to 600 nm and upgrading to a longer beam line that can accommodate grating separation of 4.5 m will potentially increase the area to ∼160 cm^2^. Another key advantage of the three PGMI is in terms of the accepted neutron flux, as the uncertainties in the NI contrast measurements are purely statistical. The neutron acceptance of a perfect crystal is orders of magnitude smaller than the broadband acceptance of the three PGMI.

One of the hallmark neutron interferometer experiments was the “COW” experiment (named for the authors of the first paper: Collella, Overhauser, and Werner), which measured the phase shift of neutrons caused by their interaction with Earth’s gravitational field [34], which is a measure of the local acceleration due to gravity “*g*.” The interferometer used had an area of ∼8 cm^2^, and the most sensitive versions of the experiment were completed with *δ*_*g*/*g*_ ≈ 10^−2^ disagreement with expectations, but with a statistical uncertainty of *δ*_*g*/*g*_ ≈ 10^−3^ [35]. Recently, it has been proposed that this disagreement may have been due to Bragg-plane misalignments in the interferometer blades [29]. Since the original COW experiments, *g* has been measured using neutrons with a very cold neutron interferometer at the 8 × 10^−4^ level [36] and a spin-echo spectrometer at the 10^−3^ level [37]. It should be noted that the current benchmark of *δ*_*g*/*g*_ = 2× 10^−11^ is set by atom interferometry [2,38,39].

The three PGMI allows for a similar experiment, where the gratings and the source slit are rotated in synchronization around the beam axis, so as to vary the angle of the diffracted path and thereby the induced gravitational potential. Considering only the current setup with 10^7^ mm^−2^ s^−1^ neutron fluence rate and the 15 by 0.5 mm slit will yield an incoming flux of *N* ≈ 7.5 × 10^7^ s^−1^. With current contrast C=0.01 and detector efficiency *η* = 0.4, the uncertainty *δϕ* in the phase (*ϕ*) due to counting statistics (shot noise) is

(5) $$\delta \phi =\frac{1}{C\sqrt{\eta Nt}}\approx 2.4\times 10^{-3}\;\;\text{rad}$$

in a *t* = 1 min measurement time. The phase due to Earth’s gravitational acceleration (*g* = 9.8 m s^−2^) is

(6) $$\phi =gT^{2}\left(\frac{2\pi }{\lambda_{G}}\right)\approx 160\;\text{rad},$$

with grating period *λ*_*G*_ = 2.4 *μ*m, and *T* = *D*_12_/*v*_*n*_ is the neutron flight time between the gratings, where *v*_*n*_ ≈ 800 m s^−1^ is the peak neutron velocity. Thus 1 min of measurement in the current setup would offer

(7) $$\frac{\delta \phi }{\phi }=\frac{\delta g}{g}\approx 1.5\times 10^{-5}.$$

Furthermore, a successful realization of the COW experiment could lead to a similar experiment to measure big “*G*,” the Newtonian constant of gravitation. The Committee on Data for Science and Technology recommended value of *G* = 6.67408(31) × 10^−11^ m^3^ kg^−1^ s^−2^ with relative standard uncertainty of 4.7 × 10^−5^ [40] consists of several discrepant experimental results. One can take advantage of the long path of the three PGMI to place a large mass along the neutron paths. The benefit over atom interferometry would be precise knowledge of the neutron path with respect to the source mass. In principle, this would allow for a measurement using the three PGMI of *δ*_*G*/*G*_ to a 10^−5^ level or smaller.

There are many aspects of the three PGMI that we can improve and expand. These include interferometer contrast, which for our setup can reach up to 32% [21]. The factors that reduce the contrast are the finite slit width, which is estimated to reduce the contrast by between 11% and 18% depending on the slit profile, the actual phase-shift profile of *G*_2_, which determines the efficiency, and neutron scattering over the long distance of the NI by air, or intervening parts such as vacuum windows. Future work will include direct assessment of individual grating diffraction efficiencies to characterize and minimize these losses. In addition to contrast gains, using a smaller grating period and increasing the interferometer length will also improve the sensitivity of the three PGMI.

We expect that the next generation of interferometers based on the three phase-grating far-field design will open new opportunities for the characterization of materials with a large autocorrelation function and for the measurement of the fundamental gravitational constants and other small forces.

## Supplementary Material

Supplemental Material

## Figures and Tables

**FIG. 1.**
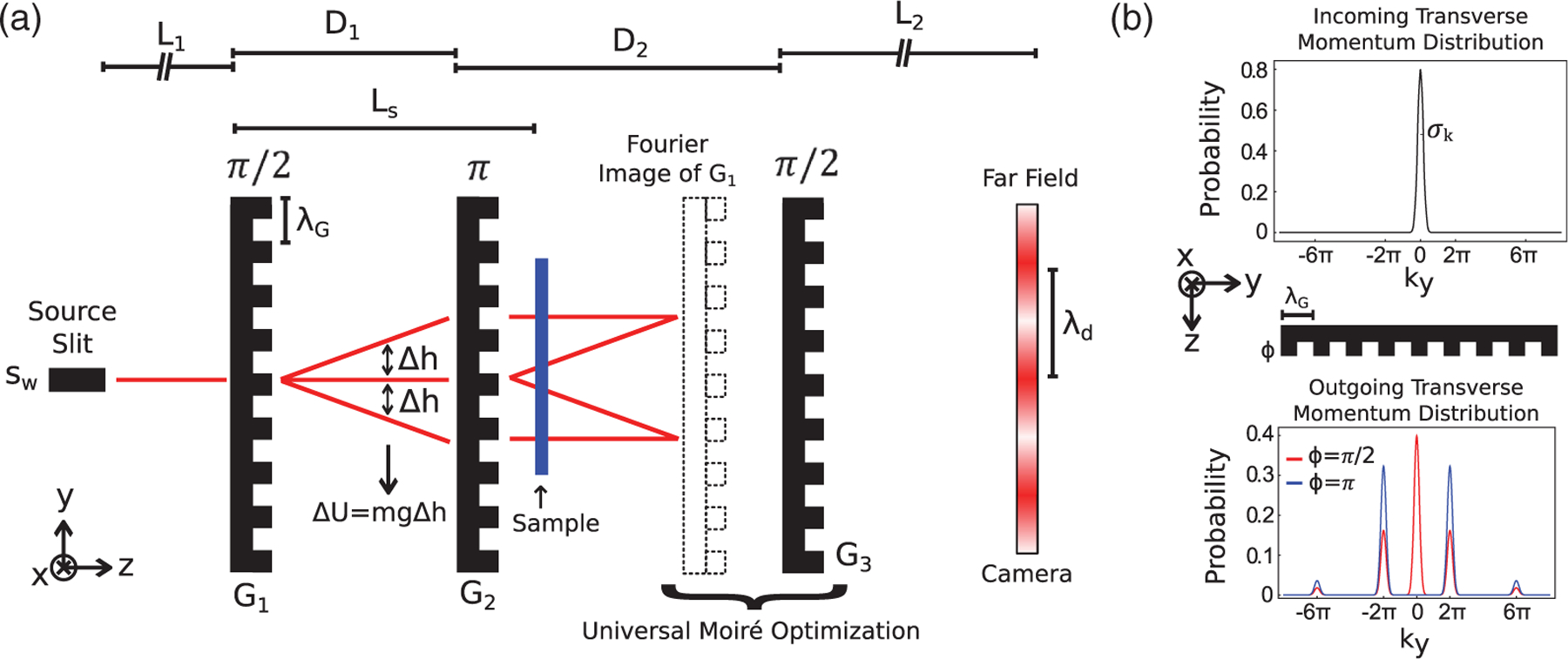
(a) Three PGMI schematic diagram where the third grating is offset from the echo plane to produce the moiré pattern, with period *λ*_*d*_, at the camera. The system can be analyzed as the superposition of continuous arrays of Mach-Zehnder interferometers, two of which are illustrated in the figure. This interferometer is sensitive to phase gradients, such as those induced by gravity. A sample may be placed between the gratings for phase and dark-field imaging. (b) Writing a phase over the transverse coherence length modifies the neutron’s transverse momentum distribution and induces diffraction. Shown is the action of 50% comb-fraction phase grating whose period “*λ*_*G*_” is equal to the transverse coherence length “*ℓ*_*c*_” of the incoming neutron wave packet, *λ*_*G*_ = *ℓ*_*c*_ = 1/(2*σ*_*k*_). For a *π* phase grating, the zeroth diffraction order is completely suppressed.

**FIG. 2.**
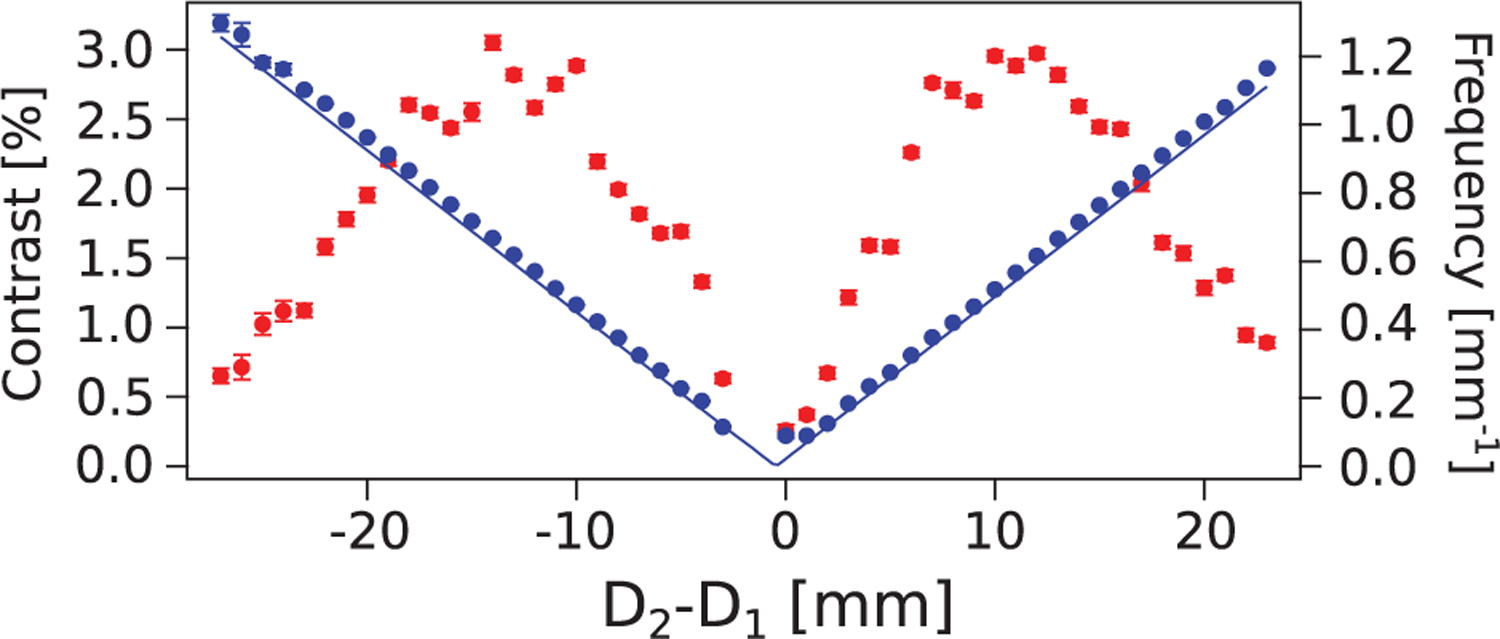
The measured contrast (red) and frequency (blue) of the interference pattern at the camera as a function of the difference between the grating separations. The uncertainties are purely statistical. The plotted theoretical frequency (straight blue line) derived from Eq. (1) shows good agreement with the measured data.

**FIG. 3.**
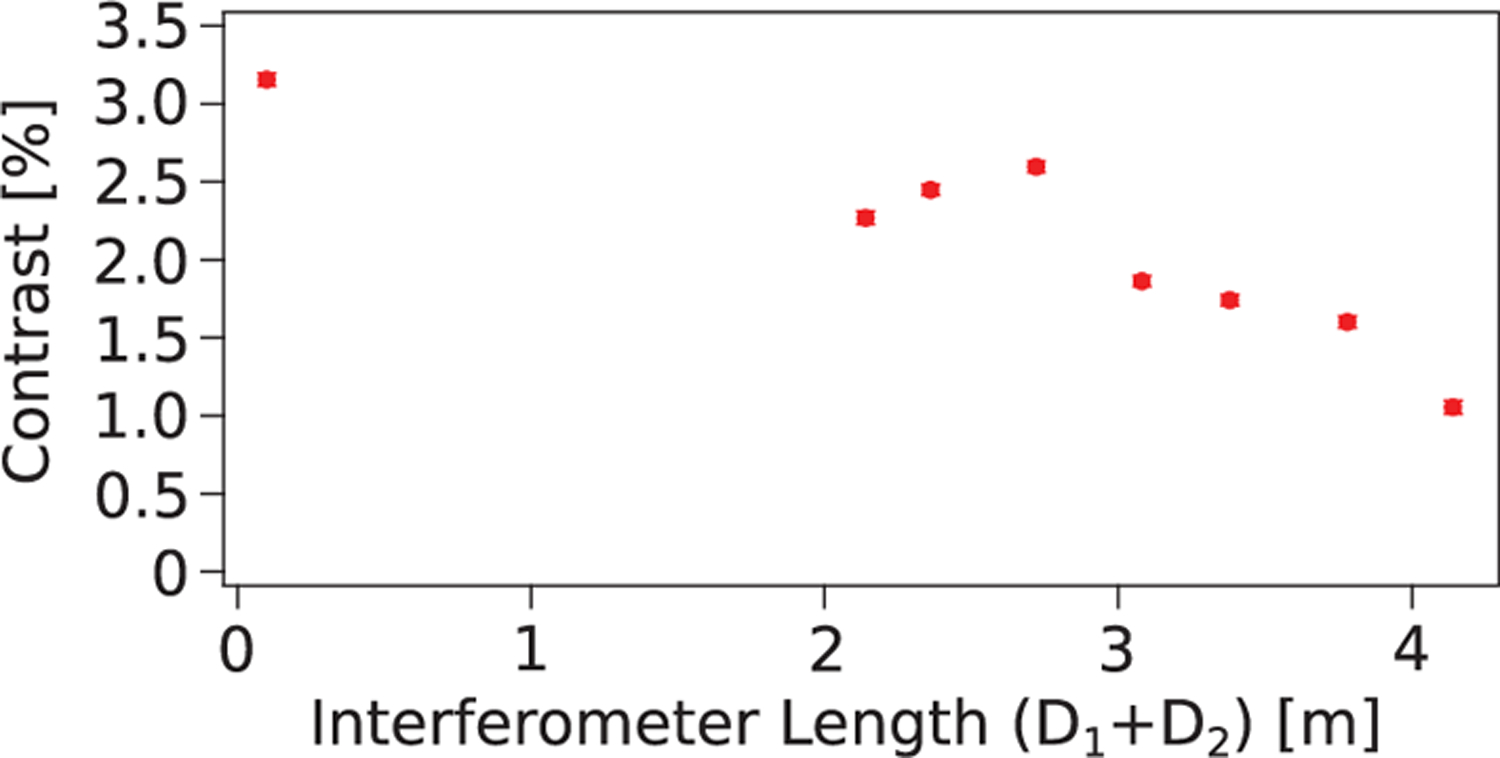
Peak contrast, where *D*_2_ − *D*_1_ ≈ 1.2 cm, as a function of the distance between the first and third grating. The purely statistical uncertainties are smaller than the individual points.

**FIG. 4.**
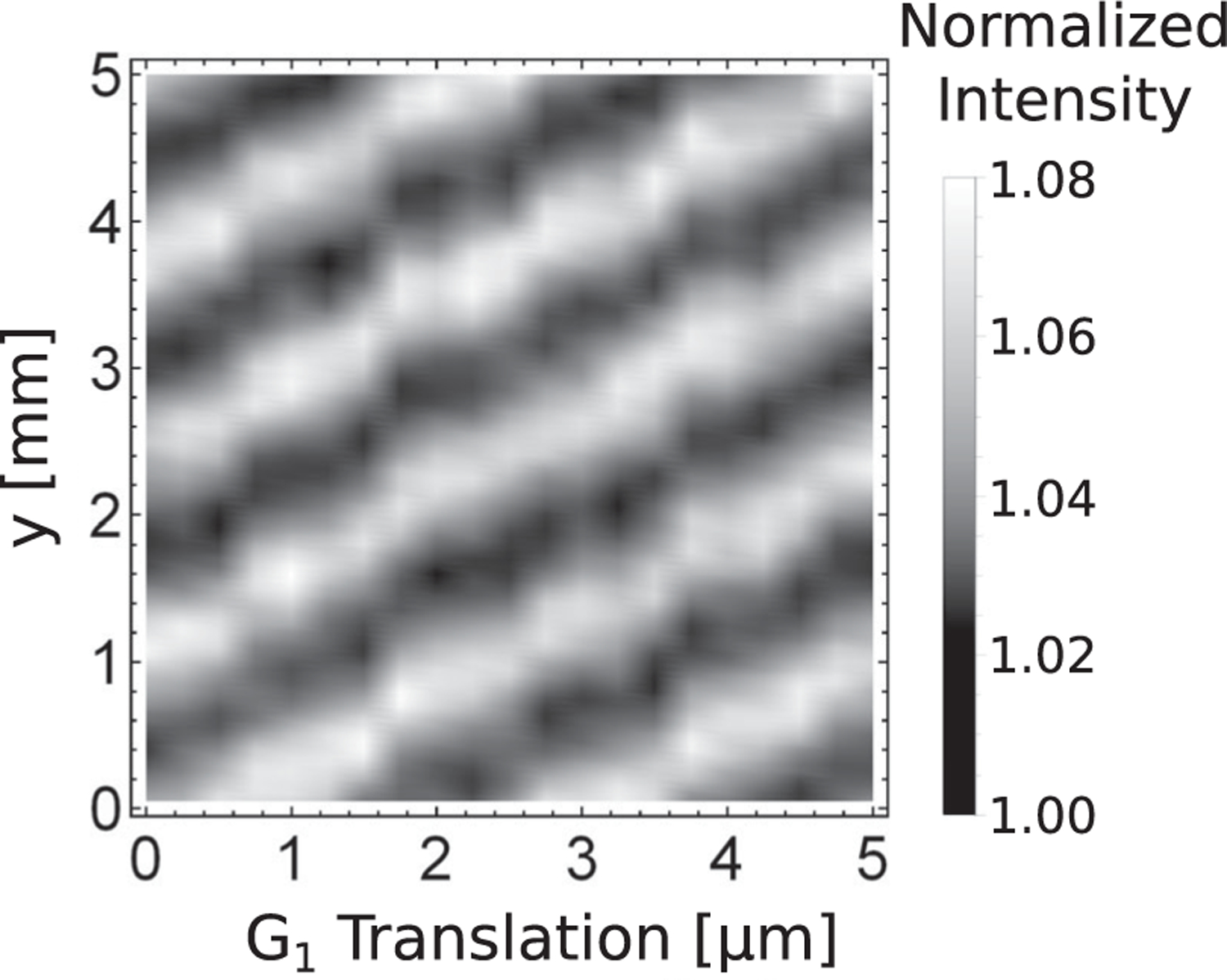
Phase stepping. The phase of the interference fringes at the detector is linearly varied by a parallel translation of the third grating. The interferometer length was set to 2 m, and *D*_2_ − *D*_1_ ≈ 1.2 cm to optimize contrast. The third grating was then translated along the grating vector [along the *y* direction in Fig. 1(a)] from 0 to 5 *μ*m, in increments of 0.25 *μ*m.

**FIG. 5.**
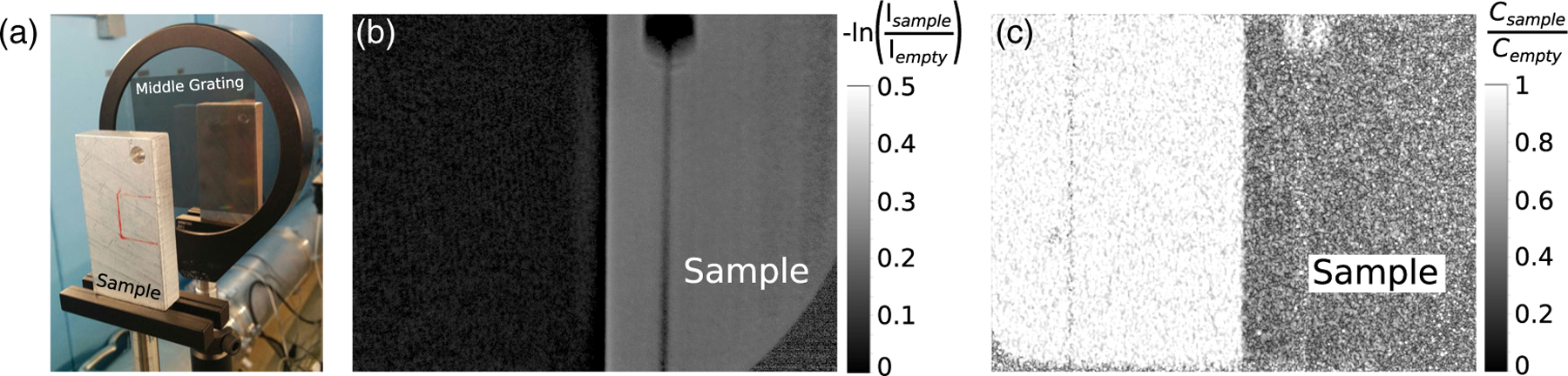
Sample imaging. (a) A rectangular sample of 6061 aluminum alloy was placed downstream from the second grating. (b) Linear attenuation of integrated intensity. The shape of the sample and the hole in the corner are recognizable in the image. (c) Normalized contrast. It is observed that the sample degrades the relative contrast to 0.28, most likely due to small angle neutron scattering off of the microstructure present in the alloy.
