# Supplementary material for: Three Phase-Grating Moiré Neutron Interferometer for Large Interferometer Area Applications
Source: Phys Rev Lett. Author manuscript; Available in PMC 2021 Dec 13. (PMC8667086; doi:10.1103/PhysRevLett.120.113201)
Supplement: Supplemental Material [file NIHMS1753140-supplement-Supplemental_Material.pdf]

I. SUPPLEMENTARY MATERIAL

The action of the phase-gratings can be understood by noting that writing a phase over the neutron coherence length modifies its momentum. The induced phase shift on a neutron due to a uniform slab of material of thickness D is:

$$\phi = -Nb_c\lambda D \tag{1}$$

where Nb_c is the scattering length density of the material (for Silicon $Nb_c \sim 2.07 \times 10^{14}$ m^{-2}), and λ is the mean neutron wavelength. The momentum operator $\hat{p} = -i\hbar\frac{\partial}{\partial y}$ suggests that a phase ramp induces a momentum shift whereas step gratings correspond to introducing momentum sidebands. Consider a neutron traveling along the z-direction with momentum $\hbar k_z$ incident onto a phase-grating as shown in Fig. 1b. The transverse wavefunction is typically assumed to be a Gaussian:

$$\Psi(y) = \left(\frac{1}{2\pi\ell_c^2}\right)^{\frac{1}{4}} e^{-\frac{y^2}{4\ell_c^2}} \tag{2}$$

where the coherence length is given by $\ell_c = 1/(2\sigma_k)$, and where σ_k is the spread of the wavepacket's transverse momentum distribution along the y-direction. The momentum distribution of the outgoing wavepacket is plotted in the lower half of Fig. 1b and it is given by

$$P(k_y) = |\mathcal{F}\{\Psi(y)e^{i\frac{\phi}{2}\mathrm{sign}[\cos(Gy)]}\}|^2 \tag{3}$$

where $\mathcal{F}$ is the Fourier transform, and $G = 2\pi/\lambda_G$ is the grating vector where λ_G is the grating period. The 1st order diffraction peaks are located at:

$$k_y = \pm G. \tag{4}$$

The three PGMI schematic diagram is depicted in Fig. 1a. In relation to the typical MZ interferometer the setup can be viewed as an "infinite" array of MZ interferometers. One pair of such virtual MZ interferometers is illustrated, where a ray from the source is diffracted by the 0th and 1st orders of the gratings into a pair of neutron paths of nearly equal lengths, thus forming a nearly closed loop between the first and the third grating. The angle between the two orders is given by:

$$\theta = \sin^{-1}\left(\frac{\lambda}{\lambda_G}\right) \tag{5}$$

Each MZ interferometer represents a pair of mutually coherent diffraction pathways through the three gratings at a specific angle from the source. For a polychromatic source, only pathways of nearly identical lengths are mutually coherent. Since phase gratings do not reduce the transmitted flux, flux conservation means that wave interference cannot produce uniform oscillations of intensity over the full field as in the ideal MZ interferometer. Instead, the multitude of interference effects sum to a spatial pattern of intensity when two conditions are met: 1. the system has an appropriate deviation from the perfect symmetry of the MZ interferometer, for example not equidistant between the gratings; 2. observation at an appropriate distance from the third grating.

For highest contrast the first and the third grating should act as $\pi/2$ phase-gratings for the mean wavelength, while the middle grating as a π phase-grating. The middle grating acts as a refocusing pulse for the diffracted neutron wavepackets from the first grating. This refocusing, conceptually similar to a spin echo, forms a series of achromatic Fourier images at specific planes downstream. The third grating then needs to be offset from this Fourier image location in order for the moiré pattern to be observed. When the separation between the first and second grating D_1 and the separation between the second and third grating D_2 are equal, the image intensity is spatially uniform and flux conservation dictates that the intensity equals the average transmitted intensity through the gratings. As D_2 is varied a beating is produced resulting in the moiré pattern at a distance which may be observed by a camera after the third grating.

An entrance slit defines the transverse coherence length of the neutron wavepackets at the grating location. In order for the neutron wavepacket to diffract, the coherence length along the grating vector direction (along

the y-direction in Fig.1a) should be at least equal to the period of the grating:

$$\ell_c = \frac{\lambda L_1}{s_w} \geqslant \lambda_{\mathrm{G}_1} \tag{6}$$

where L_1 is the distance form the slit to the first grating, and s_w is the slit width which is the slit opening along the grating vector direction. The slit height s_h, which is the slit opening along the perpendicular direction, may be increased in order to increase neutron flux, provided that the gratings are well aligned rotationally with respect to each other.

The second grating "G_2", which is ideally a π phase grating for the mean wavelength, is placed downstream so that a Fourier image of the first grating is created at a far distance as shown in Fig. 1a. The distance between the two gratings can be substantially varied while maintaining coherence in the system. A third grating "G_3" is translated around the location of the induced Fourier image to determine the optimal contrast of the fringes at the camera. When all three gratings have the same period the fringe period at the camera is given by:

$$\lambda_d = \frac{(L_1 + D_1 + D_2 + L_2)}{|D_2 - D_1|} \lambda_{G_1}. \tag{7}$$
